# The distribution pattern of critically short telomeres in human osteoarthritic knees

**DOI:** 10.1186/ar3687

**Published:** 2012-01-18

**Authors:** Maria Harbo, Laila Bendix, Anne-Christine Bay-Jensen, Jesper Graakjaer, Kent Søe, Thomas L Andersen, Per Kjaersgaard-Andersen, Steen Koelvraa, Jean-Marie Delaisse

**Affiliations:** 1 Department of Clinical Cell Biology, Vejle/Lillebaelt Hospital, Institute of Regional Health Research, University of Southern Denmark, Kabbeltoft 25, 7100 Vejle, Denmark; 2 Department of Clinical Genetics, Vejle/Lillebaelt Hospital, Institute of Regional Health Research, University of Southern Denmark, Kabbeltoft 25, 7100 Vejle, Denmark; 3 Department of Orthopedic Surgery, Vejle/Lillebaelt Hospital, Institute of Regional Health Research, University of Southern Denmark, Kabbeltoft 25, 7100 Vejle, Denmark; 4 Danish Aging Research Center, University of Southern Denmark, J.B. Winsloews Vej 9B, 5000 Odense C, Denmark

## Abstract

**Introduction:**

Telomere shortening is associated with a number of common age-related diseases. A role of telomere shortening in osteoarthritis (OA) has been suggested, mainly based on the assessment of mean telomere length in *ex vivo *expanded chondrocytes. We addressed this role directly *in vivo *by using a newly developed assay, which measures specifically the load of ultra-short single telomeres (below 1,500 base pairs), that is, the telomere subpopulation believed to promote cellular senescence.

**Methods:**

Samples were obtained from human OA knees at two distances from the central lesion site. Each sample was split into three: one was used for quantification of ultra-short single telomeres through the Universal single telomere length assay (STELA), one for histological Mankin grading of OA, and one for mean telomere length measurement through quantitative fluorescence *in situ *hybridization (Q-FISH) as well as for assessment of senescence through quantification of senescence-associated heterochromatin foci (SAHF).

**Results:**

The load of ultra-short telomeres as well as mean telomere length was significantly associated with proximity to lesions, OA severity, and senescence level. The degree of significance was higher when assessed through load of ultra-short telomeres per cell compared with mean telomere length.

**Conclusions:**

These *in vivo *data, especially the quantification of ultra-short telomeres, stress a role of telomere shortening in human OA.

## Introduction

The factors contributing to osteoarthritis (OA) have been classified into hereditary, mechanical, and age-related factors, where the latter represent the most prominent risk factor [1,2]. Aging in OA does not simply consist in wearing of the cartilage matrix, but also involves aging of the chondrocyte, the cell responsible for cartilage maintenance. Aged chondrocytes respond differently to cytokines and growth factors, exhibit discoordinated gene expression, and are dysfunctional, performing anarchic proteolysis of matrix without appropriate repair [1,2]. Such behavior is typical of cell-cycle-arrested senescent cells, a phenotype where telomere shortening is believed to be one of the critical players [3,4]. Telomeres consist of non-coding DNA protecting the ends of mammalian chromosomes. If they shorten down to a critical level, they lose their protective capability and trigger a DNA-damage response leading to cell cycle arrest and cellular senescence [5]. Interestingly, chondrocytes close to OA lesions are positive for the senescence marker senescence-associated β-galactosidase (SA β-gal), in contrast to those further away [6], and *ex vivo *expanded chondrocytes from OA cartilage show a decreased mean telomere length, which is compatible with a role of telomere shortening in OA [7].

However, a definitive conclusion about a role of telomere shortening in OA requires further validation. First, telomere lengths should be assessed in vivo and zone-specifically, rather than after expanding chondrocytes in a culture, which is likely to affect telomere length by itself. Second, there should be awareness that telomere shortening may occur by two superimposed processes [3]: (i) gradual linear shortening reflecting the number of cell divisions (that is, 'replicative' shortening); (ii) a more stochastic process, causing sudden extensive shortening of a single telomere, and induced by various stimuli, including oxidative damage (that is, 'stress-induced' shortening). The latter process deserves special attention with respect to cell senescence, because there are indications that it is the critical shortening of a few or even a single telomere, rather than a decrease in the mean length that leads to dysfunction and induces senescence [8-10]. In chondrocytes, these two processes are likely to occur to a different extent. Replicative shortening is probably less common because articular cartilage is usually considered as a post-mitotic tissue, where cell renewal is virtually absent [1]. However, a modest contribution from cell replication may be present, since the presence of putative chondrocyte progenitor cells has recently been demonstrated in human articular cartilage [11,12]. In contrast, stress-induced shortening is likely to be a common process, because oxidative stress is believed to be enhanced by cyclic compressions such as those occurring at the loading zone [13], and such oxidative stress is thought to contribute to telomere shortening also in chondrocytes [3]. Thus, it can be imagined that telomere shortening plays a role in initiation of OA even before lesions actually appear. If single critically short telomeres are the prevailing products of stress-induced telomere shortening and if they are responsible for induction of senescence, it appears essential to assess these fragments and not merely mean telomere length in chondrocytes.

In the present study we therefore characterized the distribution of these critically short telomeres in the cartilage of human OA knee with special emphasis on whether the load of ultra-short telomeres increases closer to the central lesion site. We used a newly developed Universal single telomere length assay (STELA) [8], a PCR-based method that measures the telomere length on single human chromosome arms. In addition, we measured mean telomere length by quantitative fluorescence *in situ *hybridization (Q-FISH) [14,15] for comparison, assessed senescence through counting of cells showing senescence-associated heterochromatin foci (SAHF) [16,17], and performed histopathological scoring of OA.

## Materials and methods

### Cartilage biopsies

**Figure 1:**
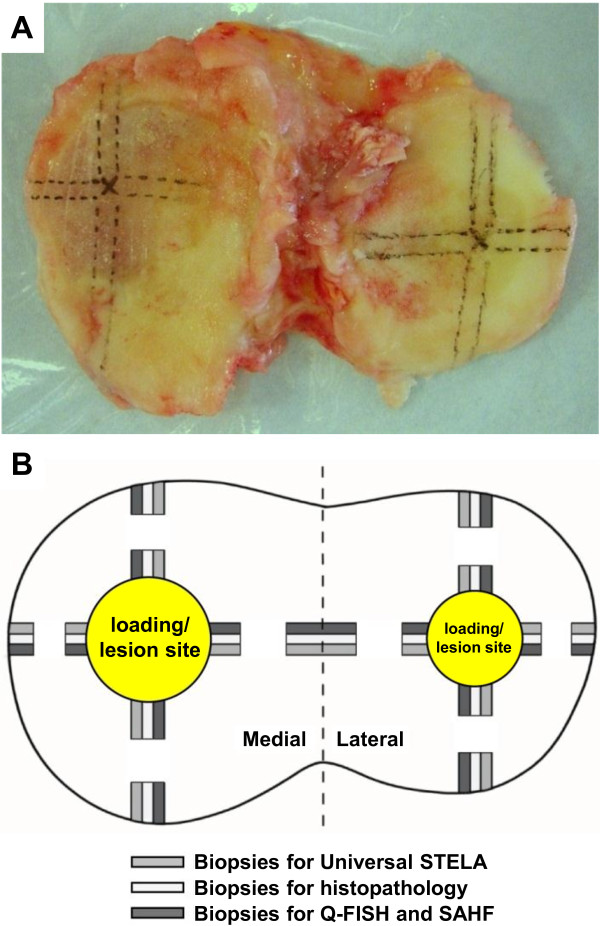
**Dissection overview of the tibia plateau from the right knee**. (**A**) After removal of the tibia plateau, markings were made with a pen prior to dissection. (**B**) Schematic drawing of sample position on the tibia plateau along the four orthogonal directions (see text). Q-FISH, quantitative fluorescence *in situ *hybridization; SAHF, senescence-associated heterochromatin foci; STELA, single telomere length assay.

**Figure 2:**
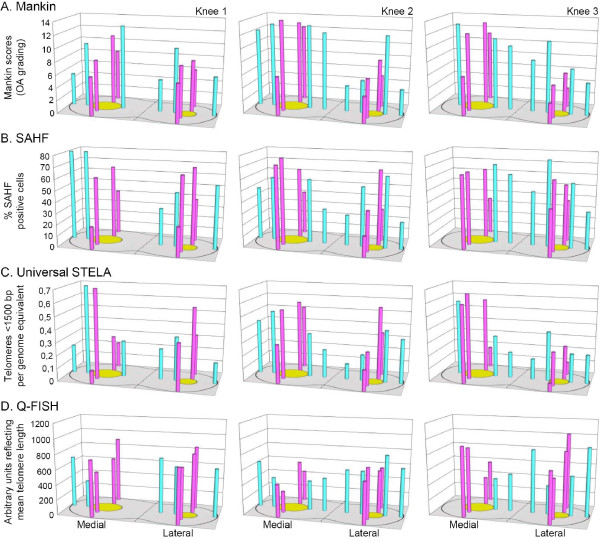
**Distribution pattern of OA scores, senescence levels, and telomere lengths around the central eroded area of the medial and lateral plateau**. OA grade (**A**), senescence level (**B**), and telomere length (**C**, **D**) were determined in biopsies taken close to and away from the central eroded area (yellow) in four orthogonal directions (as shown in Figure 1): towards the back and the front (pink bars) and towards the lateral and the medial sides (blue bars). Telomere length was assessed through Universal STELA (**C**) and Q-FISH (**D**). OA grades are expressed as Mankin scores, SAHF evaluation of senescence is shown in percentage of cells positive for SAHF per total cell number, Universal STELA data are presented as the number of telomeres below a length of 1,500 bp per genome equivalent of template DNA, and Q-FISH data are shown in arbitrary units reflecting the relative mean telomere length. Missing measurements are due to biopsies inappropriate for analysis due to too low levels of DNA or too damaged tissue. bp, base pair; OA, osteoarthritis; Q-FISH, quantitative fluorescence *in situ *hybridization; SAHF, senescence-associated heterochromatin foci; STELA, single telomere length assay.

Tibia plateaus were collected from three post-menopausal women (ages 56, 62 and 67) diagnosed with bilateral OA undergoing total knee replacement surgery (Vejle Hospital, Denmark). Collection of tissue specimens was done according to a clinical protocol approved by the Regional Research Ethics Committee of Southern Denmark (#VF20030217) and the patients signed an informed consent formula. All three patients were graded 4 according to the Outerbridge classification and 2, 2 and 4, respectively, in the Ahlback classification. None had any endocrine, metabolic or bone-related diseases. Immediately after surgery, markings were made on the two tibia plateaus to point out the exact location of biopsies (Figure 1A). Full depth cartilage biopsies were taken at two distances from the loading/lesion site along the four orthogonal directions shown schematically in Figure 1B, resulting in a total number of 48 biopsies. Each biopsy was split into three parts: one for basic histopathological characterization, one for DNA extraction followed by measurement of ultra-short telomeres using Universal STELA, and one for measurement of mean telomere length by Q-FISH and for quantification of senescence through SAHF. A few biopsies proved to be inappropriate for analysis due to too low levels of DNA or too damaged tissue (indicated in the legend of Figure 2).

### Histopathology for OA grading

Biopsies for histopathology were fixed in 3.7% neutral-buffered formalin overnight and decalcified in Idranal III solution (7% EDTA) (Sigma-Aldrich, Broendby, Denmark). Following dehydration in ethanol, they were embedded in paraffin by standard pathological procedures and serial sections of 5 μm were cut. Sections were stained with hematoxylin and Safranin O/Fast Green FCF according to standard protocols. The severity of cartilage damage was graded by two independent observers through blinded observations, by using the Histologic/Histochemical Grading System of Mankin [18].

### SAHF measurement of senescence

Tissue sections for fluorescent detection of SAHF were fixed in 3.7% neutral-buffered formalin overnight, dehydrated in ethanol and embedded in paraffin by standard pathological procedures. Paraffin-embedded tissue sections of 5 μm were cut and deparaffinized in xylene, dehydrated in ethanol and pre-treated by incubation with Proteinase K solution (DakoCytomation, Glostrup, Denmark) overnight at room temperature. Slides were washed, dehydrated in ethanol, air-dried and mounted with ProLong Gold anti-fade reagent with DAPI nucleic acid stain from Molecular Probes (Invitrogen, Taastrup, Denmark). Fluorescent images were obtained at 100 × magnification with a standard DAPI filter and were analyzed for SAHF formation after normalization of the DAPI intensity with a Laplacian filter as described by Lawless *et al. *[17]. A total of 15 to 25 cells per zone were analyzed and those showing condensation of chromatin in the nuclei were scored as positive for SAHF and were expressed as percentage of total number of cells.

### Universal STELA measurement of ultra-short single telomeres

Purification of DNA for molecular telomere measurement was performed with Blood & Tissue Genomic DNA Extraction Miniprep System from Viogene (Kem-En-Tec, Taastrup, Denmark) according to the manufacturer's manual. Briefly, the cartilage was homogenized and lysed by incubation at 60°C overnight in Viogene lysis buffer containing 4 μg/μl Proteinase K (Fisher Scientific, Slangerup, Denmark). Following incubation the enzyme was deactivated at 70°C and extraction buffer from the kit was added in a 1:1 ratio to the lysis buffer. The DNA was precipitated with 99% ethanol, washed on a silica-gel membrane (from the kit) and eluted with sterile water. Universal STELA was performed as described by Bendix *et al. *[8]. In short, purified DNA was digested by a 1:1 mixture of restriction enzyme MseI and NdeI (Medinova, Glostrup, Denmark). After digestion, annealing with a double-stranded synthetic oligonucleotide having a sticky end corresponding to MseI and NdeI digests was followed by ligation of this double-stranded oligonucleotide to the proximal end of the telomeric fragments. Next, a single-stranded oligonucleotide, with part of the sequence complementary to the telomeric overhang, was ligated to the distal end of the telomeric fragment. PCR was then performed using the two ligated oligonucleotides as targets for the PCR primers. PCR reactions were performed in a 12 μl volume containing 40 to 80 pg of ligated DNA, 1× Failsafe PCR PreMix H from Epicentre (VWR, Herlev, Denmark), 0.1 μM primers (that is, teltail and adapter) and 1.25 U Failsafe Enzyme from Epicentre (VWR, Denmark). The reactions were carried out under the following conditions: 1 cycle of 68°C for 5 minutes (fill-in step), 1 cycle of 95°C for 2 minutes, 26 cycles of 95°C for 15 seconds, 58°C for 30 seconds and 72°C for 12 minute, 1 cycle of 72°C for 15 minutes. Subsequent detection of the telomeric products by Southern blotting was carried out with TeloTAGGG Telomere Length Assay from Roche Applied Science (Hvidovre, Denmark) according to the manufacturer's manual with few adaptions. The size of the PCR products was calculated on the basis of a DIG-labeled molecular weight marker using the software VisionWorksLS Acquisition and Analysis Software from UVP (AH Diagnostics, Aarhus, Denmark). The number of bands at a length below 1,500 bp were counted, calibrated in regard to PCR template concentration and presented as the number of telomeres below a length of 1,500 bp per genome equivalent of template DNA.

### Q-FISH measurement of mean telomere length

Biopsies for Q-FISH were prepared and pre-treated as described for SAHF. Q-FISH was performed as described by Graakjaer *et al. *[15] using a fluorescent telomere-specific peptide nucleic acid probe, which binds almost stoichiometrically to the telomeres. A total of 100 ng of the probe [15] was mixed with 100 μl working solution (70 μl 70% formamide, 5 μl MgCl_2_, 10 μl MEN blocking solution (Roche Applied Science, Denmark) and 180 μl H_2_O). This mixture was added to the pre-treated slides and covered with a cover slip. Slides were then heated to 80°C for 5 minutes followed by hybridization for 60 minutes at room temperature. Subsequently, cover slips were removed followed by removal of non-specific staining and background by a short immersion of the slides in Rinse buffer (DakoCytomation, Denmark) followed by 5 minutes incubation at 62°C in Wash buffer (DakoCytomation, Denmark). Slides were dehydrated in ethanol, air-dried and mounted with ProLong Gold anti-fade reagent with DAPI from Molecular Probes (Invitrogen, Denmark). Fluorescent images of the nuclei were obtained at 100 × magnification using a standard DAPI filter and telomere images were recorded using a standard FITC filter. The intensity of telomere spots was determined by a dedicated image analysis software originally developed by DakoCytomation (Telomere Quantifier v. 1.0), followed by calculation of mean spot intensity per nucleus. A total of 20 to 30 nuclei per zone were analyzed and the mean nuclear spot intensity was calculated and expressed as arbitrary fluorescence units reflecting mean telomere length. Slide to slide and day to day normalizations proved not to be necessary probably due to the almost stoichiometric binding of the probe.

### Statistical analysis

For the paired comparison of the central and the peripheral biopsy within the same orthogonal direction we used the sign test, assuming as the null hypothesis that measurement obtained from the central biopsy of each pair is as likely to be higher as to be lower than the measurement from the peripheral biopsy. For the correlations of Universal STELA and Q-FISH measurements with Mankin scores or percentage of SAHF positive cells, we used Pearson's correlation coefficient.

## Results

**Figure 3:**
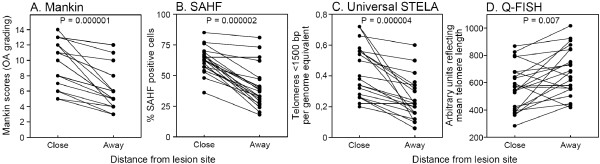
**Analysis of OA scores, senescence levels, and telomere lengths at paired loci close and away from the central lesion of the plateaus**. The measurements shown in Figure 2 were compared taking them two by two along the same orthogonal line, the one close to and the one away from the central lesion. In this way, we obtained 21 pairs of measurements for OA grade (**A**), senescence level (**B**), and relative mean telomere length (**D**), and 20 pairs of measurement for the number of ultra-short telomeres (**C**). Units are expressed as in Figure 2. *P *values obtained from the sign test are displayed. bp, base pair; OA, osteoarthritis; Q-FISH, quantitative fluorescence *in situ *hybridization; SAHF, senescence-associated heterochromatin foci; STELA, single telomere length assay.

**Figure 4:**
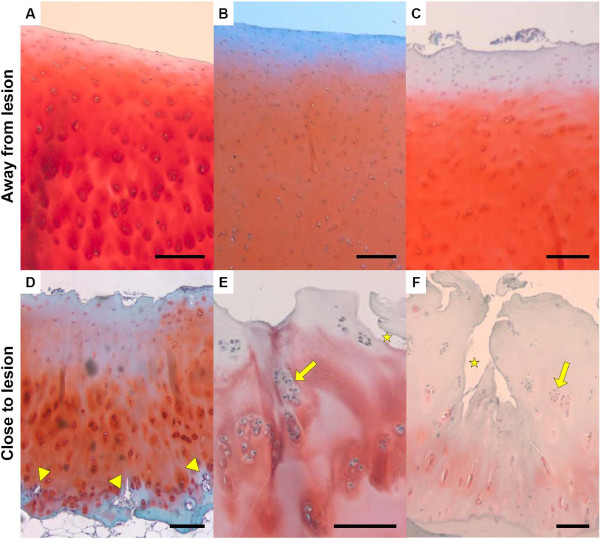
**Histological sections demonstrating typical histological appearance of cartilage at loci away **(A-C) **and close **(D-F) **to the central lesion of the plateau**. Sections were stained with hematoxylin and Safranin O/Fast Green FCF. The higher OA grades shown by the biopsies close to the central lesion (**D**-**F**) compared with those away from the central lesion (**A**-**C**) (see Figures 2 and 3), are due to changes in different histological characteristics. Away from the lesion, the cartilage appears sometimes almost intact (**A**), or shows a slight reduction in Safranin O staining (red) (**B **and **C**) sometimes along with mild surface fibrillations (**C**). These histological features are representative of early OA-induced changes. Closer to the lesion, OA is more severe as reflected by more serious surface erosion (**D**), and vascular invasion of the tidemark appears (**D**, arrow heads). Cell clusters are another typical feature observed during OA progression (**E **and **F**, yellow arrows), as well as clefts into the transitional zone (**E**, star). In severely OA-damaged tissue, clefts into the radial zone (**F**, star), more frequent cell clustering as well as extensive loss of Safranin O staining occur (**F**). Bars = 100 μm. OA, osteoarthritis.

The primary aim of this investigation was to characterize the overall distribution pattern of ultra-short telomeres in the tibial cartilage of three OA knees using biopsies from the well-defined positions shown in Figure 1. This 3-dimensional distribution pattern of ultra-short telomeres is shown in Figure 2, where their number is compared to mean telomere lengths, OA grades, and percentage of SAHF positive cells measured at the same positions. These four parameters are further analyzed in Figure 3, each along the orthogonal directions defined in Figure 1. Examples of the histological appearance of the cartilage away and close to the central lesion are shown in Figure 4.

The Mankin scores showed a highly consistent distribution pattern in agreement with daily clinical experience, namely higher Mankin scores at the medial plateau compared to the lateral plateau, and higher scores close to the cartilage lesion compared to the cartilage from the peripheral part of the joint surface (Figure 2A). The histopathological findings were consistent with the clinical grading of OA. All the paired comparisons shown in Figure 3A showed higher scores towards the central area compared to the periphery (*P = *0.000001), irrespective of the OA scores of the peripheral biopsies, which varied over a wide range. The percentage of SAHF-positive cells followed a distribution parallel to the Mankin scores (Figure 2B) supporting previous data, where SA β-gal was used as another senescence marker [6]. All 21 but 1 paired comparisons between positions close and distant to the lesion showed a higher percentage of SAHF positive cells close to the lesion (*P = *0.000002) (Figure 3B).

Overall, Figure 2C shows that the number of ultra-short telomeres exhibits the same general distribution pattern as the Mankin score and the percentage of SAHF-positive cells. Mean telomere length exhibits a similar pattern, but less clearly. When analyzing individual orthogonal directions it is obvious that the number of ultra-short telomeres is increasing when moving towards the central lesion. This phenomenon is further illustrated in Figure 3C, where it is seen that in all but one of the 20 eligible orthogonal directions, the number of ultra-short telomeres increased when moving towards the central region of the plateau (*P *= 0.000004), and the only pair of biopsies that did not show an increase showed identical values. Again, the increase was seen irrespective of the number of ultra-short telomeres in the peripheral biopsies, which varied over a wide range. When plotting the Q-FISH data in the same way (Figure 3D), the expected decline in mean telomere length when moving towards the cartilage lesion was less consistent, as it was observed in only 17 out of 21 orthogonal directions (*P *= 0.007).

**Figure 5:**
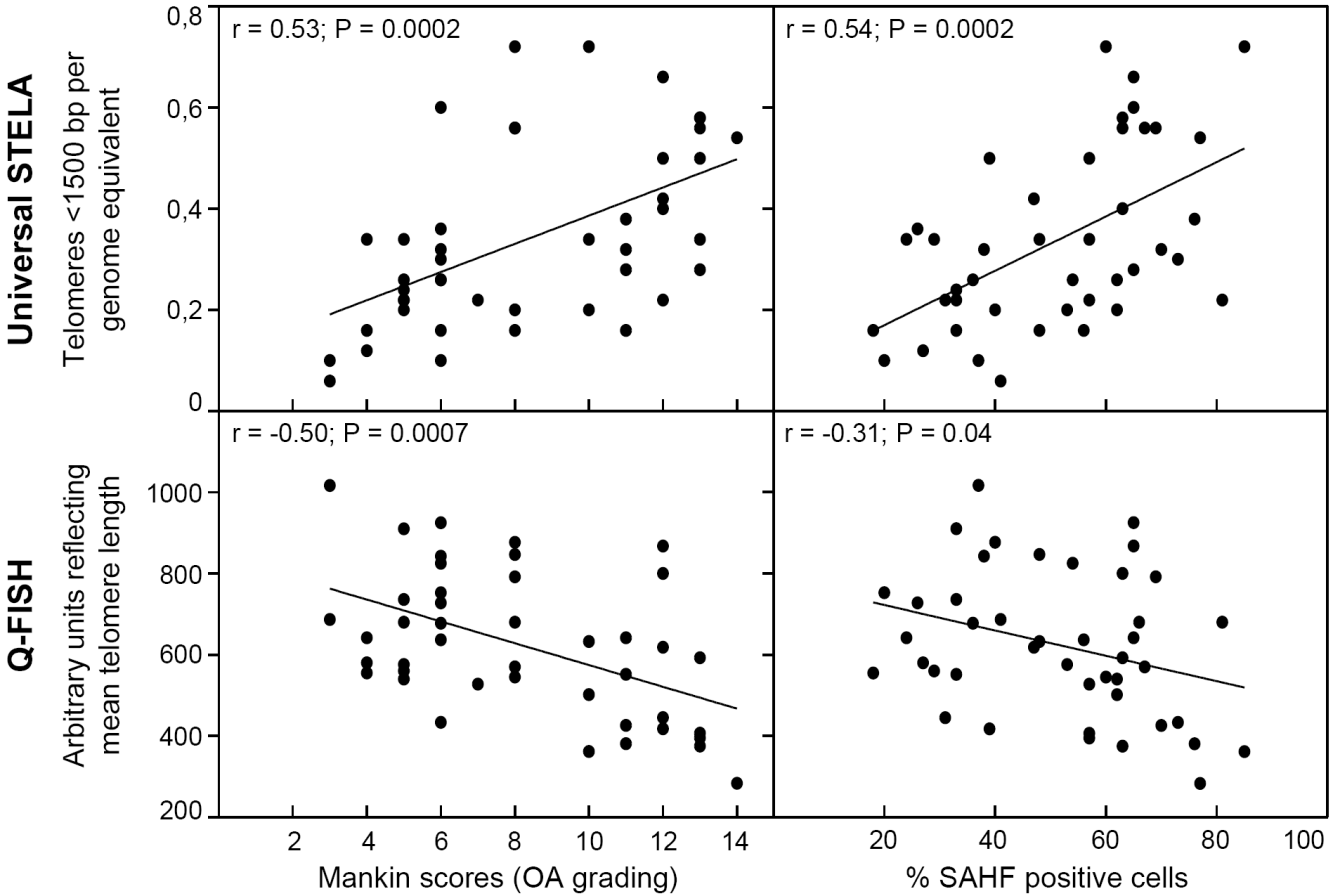
**Correlation between telomere lengths and OA grade or senescence scores**. The measurements shown in Figures 2 and 3 were used to obtain the correlation graphs of the present figure. Correlation coefficients (r) and *P *values obtained from Pearson's correlation test are displayed. bp, base pair; OA, osteoarthritis; Q-FISH, quantitative fluorescence *in situ *hybridization; SAHF, senescence-associated heterochromatin foci; STELA, single telomere length assay.

We also investigated whether telomere length directly relates with OA grade or senescence level regardless of the position where the measurements were done. Correlation graphs were therefore obtained for both the number of ultra-short telomeres and the mean telomere length against Mankin score or percentage of SAHF-positive cells (Figure 5). All these correlations were significant according to Pearson's correlation statistics. However, mean telomere lengths gave lower r values and significance levels compared with the number of ultra-short telomeres, especially when related to senescence levels.

## Discussion

**Figure 6:**
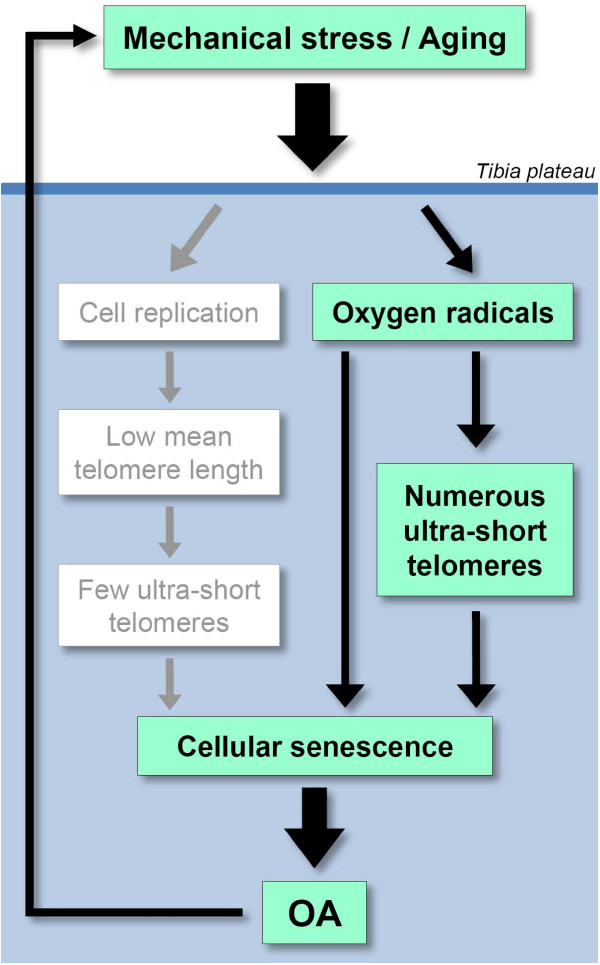
**Model for a role of ultra-short telomeres in OA**. In theory, two models for involvement of telomere shortening in OA can be considered: (i) gradual linear shortening resulting from replication for chondrocyte renewal (mainly reflected by decreased mean telomere length); and (ii) extensive shortening induced by stress factors such as oxidative damage and believed to be a key trigger of senescence (mainly reflected by the number of ultra-short telomeres). The present observations taken together with literature data strongly suggest that the latter process prevails over the former (see text). OA-induced damage is likely to cause in turn more production of oxygen radicals and cell replication, as shown by the feedback loop on the left. OA, osteoarthritis.

The findings presented in this communication support an association between OA and telomere shortening. This association had already been proposed earlier, mainly based on the analysis of *ex vivo *expanded chondrocytes [6,7], but the present assessments now demonstrate this association *in vivo *avoiding the risk of culture artifacts. Furthermore, previous evaluations were based only on mean telomere length measurements and not on the load of ultra-short single telomeres, measured here through the new Universal STELA assay. The interest in ultra-short telomeres, defined here as number of telomeres below 1,500 bp per genome equivalent, is that telomeres in this length range are believed by most researchers to contribute to the induction of senescence [19], an assumption also supported in our previously published cell culture studies, where we found a strong correlation between the number of ultra-short telomeres and the number of cells staining positive for SA β-gal [8]. Interestingly in this respect, chondrocytes close to OA lesions were reported positive for SA β-gal in contrast to those further away [6]. Similarly in the present study, the percentage of chondrocytes positive for SAHF, another senescence marker [16,17], was higher close to the lesion than further away, and this percentage correlated with both the number of ultra-short telomeres and mean telomere length. In support of an important role of ultra-short telomeres in OA development, we also found that distance to the lesion was more closely related to the load of ultra-short telomeres measured by Universal STELA than with mean telomere length evaluated by Q-FISH. Thus, when comparing telomere lengths in biopsies differing for distance to the lesion, statistical significance levels were substantially higher for analyses obtained through Universal STELA than through Q-FISH. The same holds true when comparing directly with OA grade or senescence level, regardless of the distance to the lesion. Interestingly, ultra-short single telomeres are believed to be generated through stress-induced damage, such as oxygen radicals, which are recognized as a central event in OA [3]. Taking these observations together leads to the model shown in Figure 6, where ultra-short single telomeres are suggested as likely players in OA.

The reason why we also find an association between OA changes and mean telomere length could be due to the fact that the two mechanisms of telomere shortening, that is, gradual replicative shortening and more sudden, stochastic telomere damage are not totally independent. It is likely that telomere damage due to, for example, persistent oxidative stress in the long run will lead also to a decrease in mean telomere length, which can explain why mean telomere length correlates with both distance to lesion and parallel Mankin score. This view is in line with recent reports on cultured chondrocytes showing that oxidative stress leads to a decrease in mean telomere length [20,21]. It is also interesting that the response of mean telomere length, reported by Brandl *et al.*, is not immediate but occurs gradually [20].

Our analysis is based on the variations of telomere length within restricted areas of the same cartilage tissue, that is, 20 comparisons between cells next to and away from the lesion, the latter being used as a 'control'. Further studies performed on more than three patients, could also address the inter-patient variability of the number of ultra-short single telomeres and their relation to the global OA grade of the patient. It would also be useful to extend the present study to other joints, such as the hip. Interestingly, shortening of telomeres was recently also associated with chondrocyte senescence in degenerate intervertebral discs [22,23], thereby supporting the concept that telomere shortening may be a general player in chondrocyte senescence, and not restricted to OA.

## Conclusions

Recent efforts to identify critical players in OA include elegant approaches based on gene expression profiling and gene polymorphisms [24,25]. These approaches allow very systematic screenings but miss factors such as telomere shortening, which may very well be important to take into account in a number of age-related diseases. In the present study, we used a newly-developed assay, which allows quantifying in cartilage biopsies, the minute subpopulation of ultra-short single telomeres believed to trigger cellular senescence, and we show its association with OA.

## Abbreviations

bp: base pairs; OA: osteoarthritis; PCR: polymerase chain reaction; Q-FISH: quantitative fluorescence *in situ *hybridization; SA β-gal: senescence-associated β-galactosidase; SAHF: senescence-associated heterochromatin foci; STELA: single telomere length assay.

## Competing interests

The authors have no conflict of interest to disclose in regard to this research or manuscript.

## Authors' contributions

SK and JMD have contributed to the ideas of the project. SK, JMD, ABJ, LB, JG, TLA, KS and MH have all contributed to the designing and planning of the project. MH carried out the experiments and acquisition of data assisted by ABJ, LB, JG, TLA and KS. All authors have been involved in interpretation and discussion of the results. The ethical committee protocol was written by TLA and ABJ. PKA provided the cartilage biopsies. SK, JMD and MH contributed equally to the manuscript. All authors have contributed to, read and approved the final manuscript.
